# Cynomolgus Monkeys (*Macaca fascicularis*) as an Experimental Infection Model for Human Group A Rotavirus

**DOI:** 10.3390/v10070355

**Published:** 2018-07-04

**Authors:** Gentil Arthur Bentes, Juliana Rodrigues Guimarães, Eduardo de Mello Volotão, Alexandre Madi Fialho, Cleber Hooper, Ana Carolina Ganime, Noemi Rovaris Gardinali, Natália Maria Lanzarini, Alexandre dos Santos da Silva, Jacob Pitcovski, José Paulo Leite, Marcelo Alves Pinto

**Affiliations:** 1 Laboratório de Desenvolvimento Tecnológico em Virologia, Instituto Oswaldo Cruz, Fiocruz, Rio de Janeiro/RJ 21.040-360, Brazil; juliana.guimaraes@ioc.fiocruz.br (J.R.G.); no_rovaris@yahoo.com.br (N.R.G.); natalial@ioc.fiocruz.br (N.M.L.); alexsantos@ioc.fiocruz.br (A.d.S.d.S.); marcelop@ioc.fiocruz.br (M.A.P.); 2 Laboratório de Virologia Comparada e Ambiental, Instituto Oswaldo Cruz, Fiocruz, Rio de Janeiro/RJ 21.040-360, Brazil; volotao@ioc.fiocruz.br (E.d.M.V.); amfialho@ioc.fiocruz.br (A.M.F.); carolganime@gmail.com (A.C.G.); jpgleite@ioc.fiocruz.br (J.P.L.); 3 Serviço de Controle da Qualidade Animal, Instituto de Ciência e Tecnologia em Biomodelos, Fiocruz, Rio de Janeiro/RJ 21.040-360, Brazil; cleber.hooper@fiocruz.br; 4 Virology and Vaccine Development Laboratory, MIGAL Technology Center, Kiryat Shmona 11016, Israel; jp@migal.org.il

**Keywords:** human group A rotaviruses, cynomolgus monkeys, experimental infection model

## Abstract

Group A rotaviruses (RVA) are one of the most common causes of severe acute gastroenteritis in infants worldwide. Rotaviruses spread from person to person, mainly by faecal–oral transmission. Almost all unvaccinated children may become infected with RVA in the first two years of life. The establishment of an experimental monkey model with RVA is important to evaluate new therapeutic approaches. In this study, we demonstrated viral shedding and viraemia in juvenile–adult *Macaca fascicularis* orally inoculated with Wa RVA prototype. Nine monkeys were inoculated orally: seven animals with human RVA and two control animals with saline solution. During the study, the monkeys were clinically monitored, and faeces and blood samples were tested for RVA infection. In general, the inoculated animals developed an oligosymptomatic infection pattern. The main clinical symptoms observed were diarrhoea in two monkeys for three days, associated with a reduction in plasmatic potassium content. Viral RNA was detected in seven faecal and five sera samples from inoculated animals, suggesting virus replication. Cynomolgus monkeys are susceptible hosts for human Wa RVA infection. When inoculated orally, they presented self-limited diarrhoea associated with presence of RVA infectious particles in faeces. Thus, cynomolgus monkeys may be useful as animal models to evaluate the efficacy of new antiviral approaches.

## 1. Introduction

Acute gastroenteritis (AG) is one of the leading infectious diseases worldwide, and group A rotaviruses (RVA) is one of the principal causative agents of this illness [1]. RVA vaccines are one of the most efficient strategies to reduce both mortality and hospitalisation due to severe AG [2]. Oral attenuated vaccines are developed to mimic the natural effect of infection and to prevent acute diseases [3]. Currently, two attenuated oral vaccines, one pentavalent (RotaTeq^®^, Merck & Co, Inc., Kenilworth, NJ, USA) and another monovalent (Rotarix^®^, GlaxoSmithKline Biologicals, Rixensart, Belgium), are licensed and available in several countries [3]. A review study reported a decline of 49–89% in rotavirus hospital care in children under five years in the first two years of vaccine introduction [4]. Although almost all adults have antibodies to rotavirus, they may still be susceptible to infection, and cases of reinfections are very common, due to several escape mechanisms from the host immune system and viral genetic variability [5,6]. Control of acute rotavirus gastroenteritis focuses on the treatment and prevention of dehydration, relief of symptoms and clinical signals, and restoration of normal physiological functioning [3].

Many animal models have been used to help understand the mechanisms of RVA immunity and pathogenesis, including lambs [7], calves [8], gnotobiotic piglets [9,10,11], rabbits [12], mice [13], rats [14], and recently organoids [15]. Considering that non-human primates are the most closely related animals to humans, there is a lack of information available about their susceptibility to human RVA, although they are likely to be naturally infected with simian RVA [16,17]. Although group A rotaviruses are species-specific, there is evidence suggesting the occurrence of interspecies transmission in nature and experimental infection [18,19,20]; however, compared with the vast majority of natural isolates obtained from any species, virulence of prototype Wa RVA has been associated with reduced clinical signs in heterologous hosts [21,22]. However, little has been achieved to date in attempting to develop non-human primate models for RVA infection and AG. Therefore, to establish an experimental infection model for human RVA and AG in non-human primates, it would be crucial to understand the heterologous rotavirus infection in order to conduct future vaccine trials and immunotherapy tests. It is important to emphasise that previous studies were performed on neonatal and infant monkeys [23,24,25,26], translating the importance of RVA infection in patients under 5 years old. However, studies about the pathogenesis of RVA infection in adult patients are neglected by other authors due to the reduced incidence of clinical symptoms in infected persons, but this represents an important factor to maintaining the virus in the environment [27].

In the present report, we describe a successful experimental infection of adult cynomolgus monkeys (*Macaca fascicularis*) using Wa RVA prototype to evaluate the susceptibility and pathogenesis of this species as a model for RVA infection and AG. In our hypothesis, RVA infection in adult cynomolgus monkey presents a similar gastroenteritis pattern to that observed in hospitalized adult patients: the severity of enteric disease is variable, depending on the individual immunological background [28], also reported as RVA nosocomial infection in adult patients [29]. Additionally, other authors suggest that caregivers (mother/grandmother/older siblings) may get infected through young children or may act as carriers for transmission, reinforcing the role of adult patients [27]. Also, immunodeficient adults have a higher risk of onset of symptoms and prolonged dissemination of the virus, reaching a year or more, thus representing a powerful reservoir of infection; a fact already described in immunodeficient children [30,31].

## 2. Materials and Methods

### 2.1. Animals

Nine clinically healthy male cynomolgus monkeys, with ages ranging from 32 months (2 years 8 months) to 105 months (8 years 9 months), weighing from 2.35 to 6.72 kg (Table 1), were screened for RVA by serological and molecular assays during a quarantine period. All animals were obtained from the Primatology Department of the Institute of Science and Technology in Biomodels (ICTB) of the Oswaldo Cruz Foundation (Fiocruz), Rio de Janeiro, RJ, Brazil at Animal Biohazard Level 2 facilities during quarantine and throughout the whole experiment. Animals were individually housed in stainless steel squeeze-back cages (0.77 m height × 0.60 m width × 0.68 m depth) in climate-controlled rooms (temperature of 23 ± 1 °C and humidity 55 ± 5%) with a 12h light/dark cycle and fed daily with commercial primate diet supplement, fresh fruits and vegetables. Water was provided ad libitum. Housing standard adopted in our study adhered to space recommendations for individually non-human primates with a maximum weight of 7 kg, in accordance with the Brazilian Normative Resolution CONCEA n.28, of 13 November 2015 (http://www.mct.gov.br/upd_blob/0240/240230.pdf). The origin of the primate colony and animals’ maintenance were previously described [32]. All animals had health certificates, which guaranteed the absence of infectious diseases, and were confirmed to be seronegative for RVA immunoglobulins by in-house immunoassay. A serological survey confirmed that they were free of simian immunodeficiency virus (SIV) and simian type D retrovirus (SRV/D) [33]. Cynomolgus monkeys were used in this study because of our expertise with *Macaca fascicularis* used in experimental infection [34,35,36].

Fiocruz Ethics Commission for the Use of Animals approved the experimental protocol on 26 September 2011 (CEUA-Fiocruz LW-35/11). Monkeys were monitored by veterinarians and technicians in procedures and daily for ataxia, dehydration and weight loss. If any monkey developed severe gastroenteritis, in which there was significant decrease in weight and ataxia, this animal would be euthanized to reduce suffering/pain. Clinical procedures were performed under anaesthesia, and all efforts were made to minimize painful procedures. The study protocol was conducted in strict accordance with the recommendations from the Guide for Care and Use of Laboratory Animals of the Brazilian Society of Science in Laboratory Animals (SBCAL) and the National Council for the Control of Animal Experimentation (CONCEA, Brazil).

### 2.2. Virus Inoculum

Human RVA was used as virus inoculum (RVA/human-tc/USA/Wa/1974/G1P[8]), inoculated in cell culture using monkey African green kidney cells at passage #58 (MA-104, ATCC^®^ CRL-2378). After viral cytopathic effect observation, virus purification was performed with caesium chloride gradient as described previously [37,38]. Virulent RVA for inoculation of cynomolgus monkeys was used at dose 3.1 × 10^6^ fluorescent focus-forming units (FFU)/mL [39].

### 2.3. Experimental Design

Seven animals (named as X9, AB7, V11, Z7, U13, T7, and AA7) were inoculated orally with 2.0 mL (3.1 × 10^6^ FFU/mL) of RVA suspension diluted in isotonic saline solution (inoculated group), and two animals (V7 and X11) were administrated an isotonic saline solution orally and maintained as uninfected controls (placebo group). Administration was carried out in the stomach by an appropriated gavage tube. Before viral inoculation, serum and faecal samples were collected to establish individual baseline values for biochemical, virological and immunological parameters. Clinical signs of diarrhoea, vomiting, ataxia, and dehydration and body temperature were checked daily. Watery or semiliquid faeces were considered diarrhoea, and a severity scoring of diarrhoea was adopted (mild, moderate, and severe). Monkeys were considered febrile when their body temperatures were ≥38.5 °C. Faecal samples were collected daily from 1 to 10 days post-inoculation (dpi). To collect blood samples and check their weights, animals were anaesthetised on 1, 3, 7 and 10 dpi as described previously [34]. On 10 dpi, all animals were euthanized by total exsanguination under deep anaesthesia according to previous experimental infection protocol [34].

### 2.4. Haematological and Biochemistry Analyses

Blood samples were drawn from the femoral vein. For haematocrit ranges and leukocyte counts, the analysis was performed by a fully automated veterinary haematology analyser, pocH-100iVDiff (Sysmex Europe GmbH, Hamburg, Germany). For different leukocyte ranges, a series of blood films were prepared and stained using May–Grünwald–Giemsa staining. Blood samples were centrifuged at 3800× *g* for 10 min, and sera aliquots were collected and stored for biochemistry analysis (potassium, chloride and sodium). Analyses were performed using Vitros 250 (Ortho Clinical Diagnostics–Johnson & Johnson, Auckland, New Zealand). Haematocrit, biochemistry, and leukocyte parameters were based on previous study with cynomolgus monkeys maintained under laboratory conditions [40]. However, levels from all animals were below the parameters even before inoculation, and therefore pre-inoculation levels of the animal itself were used as parameter. The non-human primates breeding colony from Fiocruz was established and has had no new genetic improvement since 1980 until now; thus, the haematology base-line values are particular. Additionally, tropical environment conditions induce different values than other colonies.

Statistical analyses were conducted using GraphPad Prism version 7.0 (GraphPad Company, San Diego, CA, USA). One-way ANOVA was used for intra group analysis, and paired Student *t*-test was used for inter groups’ analyses. Differences were considered significant at *p* ≤ 0.05.

### 2.5. Human Group A Rotavirus Detection by Enzyme Immunoassay

Faecal samples were collected and screened for detection of RVA by enzyme immunoassay (EIA) RIDASCREEN^®^ Rotavirus (R-Biopharm AG, Darmstadt, Germany) following manufacturer’s recommendations.

### 2.6. Qualitative and Quantitative Human Group A Rotavirus RNA Detection by Molecular Amplification Procedures

RNA was extracted from 10% of faecal suspensions and sera samples using QIAamp Viral RNA Mini Kit^®^ (Qiagen, Valencia, CA, USA) following manufacturer’s protocol. The cDNA extension was performed using High Capacity cDNA Reverse Transcription Kit^®^ (Applied Biosystems/Life Technologies, Carlsbad, CA, USA) using recommended primers and protocol to amplify *VP6* gene [41].

To confirm the virus infection, a quantitative assay was performed, and viral RNA was quantified by real time polymerase chain reaction (qPCR) with the TaqMan^®^ Universal PCR Master Mix (Applied Biosystems/Life Technologies). The qPCR was performed using NSP3-specific primers and a TaqMan^®^ Probe (Applied Biosystems/Life Technologies) as previously described [42], using Applied Biosystems 7500 Real-Time PCR System (Applied Biosystems, Foster City, CA, USA). Assays were run in duplicate and used a calibrated standard curve generated with serial dilutions (ranging from 10^0^ to 10^7^) of a plasmid clone characterized previously [43]. Samples that showed signals crossing the threshold line in both replicates until Ct ≤ 40 and presenting a characteristic sigmoidal curve were considered positives. Target copy numbers were calculated based on Ct values in reference to the standard curve. The number of copies per millilitre was determined by adjusting values according to volumes used for each step of the procedure (i.e., extraction, and the qRT-PCR reaction). Limit of detection for RTqPCR assay is of 4.4 × 10^2^ RNA copies/mg or mL [44].

### 2.7. Evidence of Infectious Particles from Faeces

Stool samples of inoculated and control animals were used to inoculate MA-104 cells to confirm RVA infectivity. One faeces sample of each animal was selected by positive RVA RNA in PCR, in different times of infection, to be used as inoculum: i.e., AB7 at 5 dpi; T7, V11 and Z7 at 7 dpi; AA7, X9, U13, V7 and X11 at 6 dpi. Cells infection was performed as described previously [39]. In brief, monolayers of cells were grown in Lab-Tek^®^ Chamber Slide™ System (Nalgene Nunc, Naperville, IL, USA), and infection was performed with supernatant of 10% of faeces suspensions treated with trypsin to activate the virus. In order to clarify and remove bacteria, inoculum was centrifuged previously at 10,000× *g* for 15 min and filtered by a 0.45 µm filter.

After 18 h incubation of the culture, indirect immunofluorescence was performed with adaptions of a previous protocol described by Montero et al. (2008) [45]. Cells were fixed with 4% paraformaldehyde. Permeabilization was proceeded by incubation in 0.5% Triton X-100 with 1% bovine serum albumin (BSA), and blocking was carried out by incubation with 4% BSA, 0.1% tween 20. Cells were incubated with primary antibodies (MAb anti-VP6 IgG; Abcam^®^, Cambridge, UK), followed by incubation with corresponding secondary antibody (donkey anti-mouse IgG/Alexa Fluor^®^ 488; Abcam^®^, Cambridge, UK), both incubation were carried out for 30 min at room temperature. Slides were finished using glycerol, and then covered with glass slides. Cells were analysed using ApoTome microscope (Zeiss, Jena, Germany).

## 3. Results

**Figure 1:**
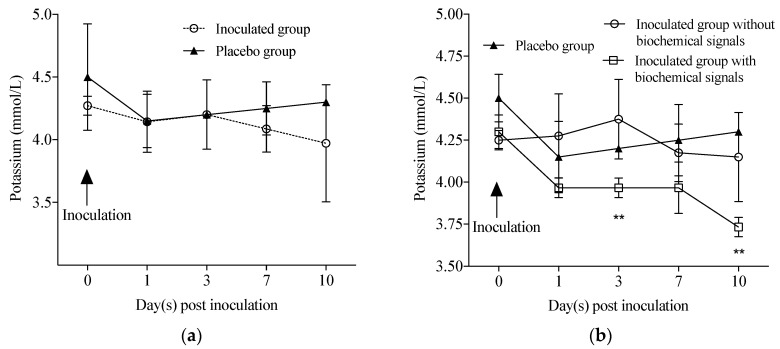
Sera potassium levels of cynomolgus monkeys. (**a**) Differences between the inoculated group and the placebo group. The arrow represents the inoculation time; (**b**) Comparison among the inoculated group without biochemical signals (Z7, V11, X9 and U13); inoculated group with biochemical signal (potassium decrease–T7, AB7 and AA7); and placebo group (V7 and X11). The arrow represents inoculation time. ** Statistical analyses between groups presented significance in reducing of K^+^ levels at 3 and 10 dpi (*p* < 0.01).

Clinical signals of RVA infection, such as fever (≥38.5 °C), emesis, anorexia, and weight loss, were investigated during this study. The primary symptom of RVA infection was diarrhoea observed during the course of infection (1 to 10 dpi); exceptions were T7 and AB7 monkeys, which presented episodes of diarrhoea for three days (from 7 to 9 dpi) and two days (from 2 to 3 dpi), respectively. In T7, diarrhoea severity was classified as moderate on the first two days and mild at the last one. In AB7, diarrhoea was severe for two days. Analysis of biochemical parameters in the sera showed a decrease in the potassium (K^+^) levels from 7 to 10 dpi in the inoculated group, but no statistical significance was observed (Figure 1a). Such as three inoculated monkeys (T7, AB7, and AA7) showed a decrease in serum K^+^ levels from 1 to 10 dpi, which two had diarrhoea. Thus, the inoculated group was divided into animals with and without biochemical signals. According statistical analysis, at 3 and 10 dpi, the inoculated group with biochemical changes K^+^ levels was significantly reduced in comparison with placebo group (*p* < 0.01) and inoculated group without K^+^ decrease (*p* < 0.05) (Figure 1b). Sodium (Na^+^) and chloride (Cl^−^) levels as well as specific haematological counts did not change in the inoculated group. An elevation in the lymphocyte count was observed in one animal, AB7, from 7 dpi onwards (5.6 × 10^3^/mL).

**Figure 2:**
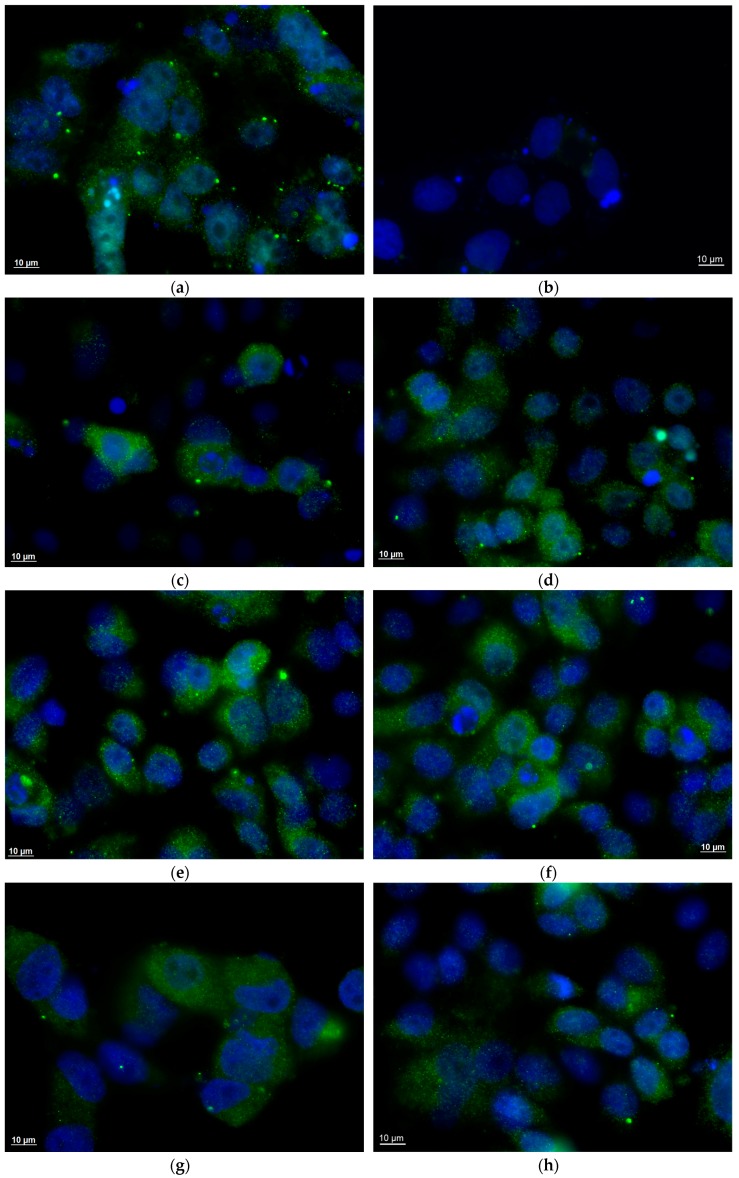

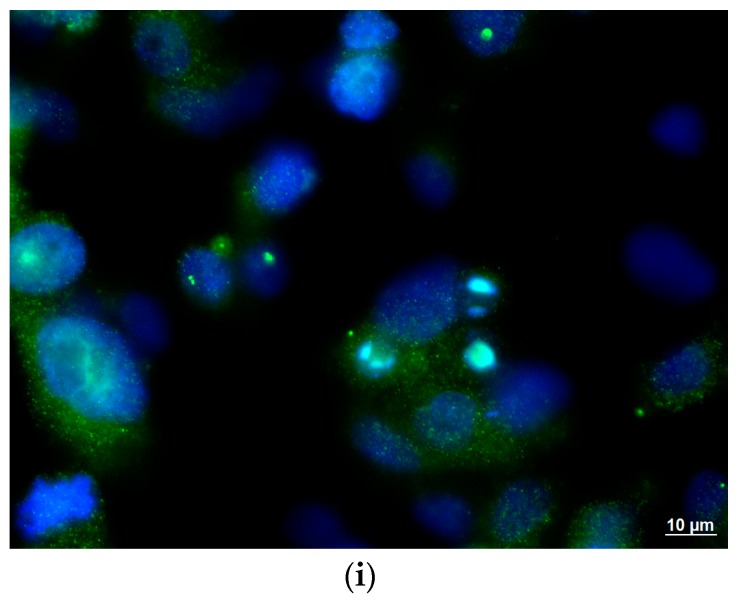
Indirect immunofluorescence of MA-104 cell culture infected with Wa rotavirus (RVA) of inoculum and monkeys’ faeces, showing rotavirus infection pattern with granular and cytoplasmatic fluorescence in cell monolayer. (**a**) RVA inoculum administrated in monkeys (3.1 × 10^6^ FFU/mL). (**b**) V7 monkey faeces at 6 dpi (control monkey). (**c**) T7 monkey faeces at 7 dpi. (**d**) U13 monkey faeces at 6 dpi. (**e**) Z7 monkey faeces at 7 dpi. (**f**) X9 monkey faeces at 6 dpi. (**g**) AA7 monkey faeces at 6 dpi. (**h**) AB7 monkey faeces at 5 dpi. (**i**) V11 monkey faeces at 7 dpi. Wa RVA antigen (VP6) was stained in green (Alexa Fluor^®^ 488 nm) and nucleus were stained in blue with DAPI (4′,6-diamidino-2-phenylindole).

The RVA replication in the enterocytes of cynomolgus monkeys occurred through intermittent viral RNA detection in the faeces and a short-term detection of RVA RNA in the serum (Table 1). Monkeys’ weight shown in Table 1 was measured at 3 dpi, and no significant difference of weight loss was observed from the first until the last day. Besides the detection of infectious particles in the faeces of all infected monkeys, there was also demonstrated viral replication in MA-104 cells. Specific fluorescence was only visible in the cytoplasm of infected cells, showing a granular fluorescence pattern as presented in Figure 2. These findings were not necessarily accompanied by episodes of diarrhoea. Three animals, Z7, T7 and AA7, were considered febrile, presenting temperatures of 38.6 °C (7 dpi), 38.5 °C (1 dpi), and 39.0 °C (1 dpi), respectively. No other animal had fever episodes during the whole experiment. In control animals, the average temperature presented was 36.9 °C.

All inoculated monkeys in the inoculated group showed the elimination of RVA in faeces. The RVA shedding in faecal samples showed an intermittent pattern of viral elimination. The first viral RNA detection occurred between 1 and 3 dpi and persisted from 7 to 10 dpi (Table 1). Monkeys in the inoculated group showed an interval of one to two days without RVA RNA detectable in their stool samples; five of them (AB7, T7, X9, AA7 and Z7) had this interval twice (Table 1). During the course of viral infection, the animals presented an average of 10^3^ RNA copies/mg in their faeces. The presence of infectious viral particles in faecal samples at different days post RVA inoculation occurred independently if monkeys presented diarrhoeal episodes or not (Figure 2). No evidence of infectious particles was detected in the faeces of the two control animals. Viral RNA in serum was detected in five out of seven animals from the inoculated group on either 1 or 3 dpi and occurred for only one day in four monkeys; the exception was Z7, for which viral RNA was detected on both days (Table 1). All samples were negative for RVA by EIA.

## 4. Discussion

In this study, we reproduced an infection with heterologous RVA in cynomolgus monkeys. Human RVA is an important causative agent of AG and death in children under 5 years of age [1]. Human RVA-induced AG was accompanied by virus shedding in juvenile (AB7-2 years and 8 months) and adult (T7-8 years and 9 months) monkeys. Most of the inoculated animals developed oligosymptomatic infection, which corroborates a previous report showing an effective Wa RVA infection in 4/6 inoculated new-born monkeys, with one presenting diarrhoea [24]. Leong et al. [25] showed a dose-dependent pattern of RVA infection and AG in new-born cynomolgus monkeys (24 h old). The new-born susceptibility to human RVA was also described in rhesus macaques, vervet monkeys and baboons [23,26]. Even infected new-born rhesus monkeys with homologous wild-type RVA may not show signs of diarrhoea or dehydration and remain clinically normal despite shedding large amounts of RVA in their faeces [46].

In our study, RVA detection was assessed in faeces during the whole period, whereas in the placebo group all samples were negative. The first viral RNA detection in each animal occurred at 1, 2 or 3 dpi. After a one- or two-day interval without viral shedding, a second detection was observed, occurring either on 4 or 6 dpi. Thus, the first period of viral excretion most likely represents part of the inoculum (RVA incubation period after oral inoculation) being naturally shedding, whereas the second wave of viral shedding observed (second detection) is likely indicative of the replication. Comparatively, after the incubation period, monkey AB7 showed continuous viral shedding (5 days), whereas monkey U13 shed for just 1 day. Similarities in this profile were observed in human volunteers, with RVA induced-illness occurring 2–6 days after viral ingestion and continuing for 1–4 days, as reviewed by others [47]. Rotavirus shedding continued for up to 10 days after viral ingestion in humans [48]. All RVA inoculated monkeys presented an intermittence in rotavirus shedding, interestingly four monkeys (AB7, AA7, X9, and T7) showed a third detection period. In our opinion this last period detection proves rotavirus replication. This intermittent rotavirus shedding occurs in both symptomatic and asymptomatic children [49]. Despite detection of rotavirus RNA in faeces, the viral load was not elevated, with an average of 10^3^ copies of RNA/mg, and the highest quantification was of 10^4^ copies of RNA/mg. Adults eliminate less rotavirus in faeces than children [50].

We detected viral RNA in sera from 5/7 inoculated animals. Control animals were negative for WA RVA RNA during the whole period of study. This detection was transient (one or two days in the majority of inoculated animals). Comparatively, RVA antigen and RNA were also detected in sera of 50 to 90% of children with RVA infections [51,52,53,54,55]. Despite reduced clinical relevance in our study, the presence of viral RNA in the blood stream represents the capacity of the virus to pass through the intestinal barrier, confirming virulence of human RVA in cynomolgus monkeys. Additionally, detectable RVA viraemia may contribute to the extraintestinal spread of the virus during the intestinal infection phase [31,51,52,56,57,58,59]. RVA most likely escapes the intestinal tract in children and accesses the bloodstream through M cells, which overlie Peyer’s patches [51]. Although monkey T7 presented expressive AG, it did not develop viraemia, indicating that AG may not be the critical determinant of antigenaemia or viraemia [52,60].

EIA failed to detect RVA antigens in sera or faeces of RVA-inoculated cynomolgus monkeys, confirming other previous and inconclusive studies about juvenile and adult non-human primate susceptibility [24,25]. Recently, more sensitive molecular methodologies have improved human RVA diagnosis [61,62]; thus, we confirmed positive results by RT-PCR and qPCR [63]. The commercial antigen-based-test (EIA) has a minimum limit of detection of 10^6^ viral particles [64]. Other authors reported that symptom-free adult monkeys can shed RVA in quantities so low as to be undetectable by most routine assays [65]. In our opinion, a reduced viral load observed in cynomolgus host justified the absence of RVA antigen detection in this model, as observed in other heterologous infections [32,34]. In order to confirm that the virus detected in faeces was not merely free RNA, but the complete infectious particles (virion), the infection of MA-104 cells with faecal samples was performed. The presence of infectious particles in faeces was confirmed in seven inoculated animals. Faecal samples corresponded to 5, 6 and 7 days post inoculation, the second period of viral shedding in faeces, probably the product of RVA replication in enterocytes of inoculated cynomolgus monkeys.

The finding of progressive low serum concentration of K^+^ detected in T7, AB7 and AA7 from 1 dpi onwards was coincident with episodes of diarrhoea diagnosed in two of these animals during experimental infection, and similar findings were described by other authors [66,67]. Commonly, diarrhoea pathogeny includes water and nutrient absorption reductions with an elevation of intestinal fluid secretion, consequently resulting in electrolyte and water loss [68]. However, no changes were observed in Na^+^ and Cl^−^ levels and dehydration signs in our animal model; this clinical pattern may be also observed in children with gastroenteritis [69,70]. In general, RVA AG infection is characterized as an inflammatory intestinal disease localized in the upper intestinal tract, without demonstration of severe systemic effects [71]. Detected viraemia observed in our study was not associated with diarrhoea severity and/or biochemical blood changes in RVA infection. Elevation of lymphocyte counts was the other haematological change identified in AB7 from 7 to 10 dpi; a short-term T cell elevation has also been described in human rotavirus infection in gnotobiotic pigs [72]. Viral infections were associated with increase of lymphocytes levels, and this was also observed for rotavirus [73]; however, in our study, lymphocyte variation was insignificant. Although viral infection has developed an oligosymptomatic gastroenteritis pattern in some animals, the decision to use a heterologous rotavirus in the establishment of an experimental infection model is justified by the further model test of human vaccines and immunotherapies. A future immunotherapy could benefit especially hospitalized adult patients and immunocompromised adults with severe clinical conditions with rotavirus gastroenteritis.

## 5. Conclusions

Taken together, these results confirm that cynomolgus monkeys in different ages developed heterologous infections with human RVA. In general, cynomolgus monkeys developed oligosymptomatic pattern of infection, with episodes of AG that may have induced electrolytic disturbances with detectable RVA RNA in sera and low-titered viral shedding, but infectious, in their faeces. Other genotypes of rotavirus have emerged as a form of escape of the host immune system, thus, vaccines need to be reformulated with new genotypes. Hence, cynomolgus monkeys may represent a promising model to evaluate new candidates for human RVA vaccines, efficacy of new antiviral therapies, and studies about pathogenesis of human RVA infection.

## Figures and Tables

**Table 1:** Quantification of group A rotaviruses from faeces and sera of cynomolgus monkey by reverse transcription–real time polymerase chain reaction.

	Placebo Group Monkeys		Inoculated Group Monkeys						
	X11	V7	AB7 *	AA7	Z7	X9	V11	U13	T7 *
**Age**	5 y 8 m	6 y 10 m	2 y 8 m	3 y 5 m	4 y 6 m	5 y 9 m	6 y 8 m	7 y 2 m	8 y 9 m
**Weight (kg)**	6.29	6.72	2.43	2.35	3.98	4.66	6.70	5.83	5.19
**DPI**	Faeces (RNA copies/mg)								
**0**	(-)	(-)	(-)	(-)	(-)	(-)	(-)	(-)	(-)
**1**	(-)	(-)	(-)	(-)	(-)	(-)	(-)	6.137 × 10^3^	5.709 × 10^3^
**2**	(-)	(-)	^†††^ 3.0 × 10^3^	5.4 × 10^3^	5.691 × 10^3^	(-)	6.5 × 10^6^	4.646 × 10^3^	1.749 × 10^3^
**3**	(-)	(-)	^†††^ (-)	1.041 × 10^4^	5.211 × 10^3^	3.531 × 10^3^	3.6 × 10^3^	4.594 × 10^3^	8.229 × 10^3^
**4**	(-)	(-)	4.714 × 10^3^	3.669 × 10^3^	(-)	4.269 × 10^3^	1.068 × 10^4^	(-)	(-)
**5**	(-)	(-)	3.686 × 10^3^	(-)	(-)	(-)	(-)	(-)	(-)
**6**	(-)	(-)	3.12 × 10^3^	1.174 × 10^4^	4.217 × 10^3^	2.160 × 10^3^	9.909 × 10^3^	8.674 × 10^3^	4.251 × 10^3^
**7**	(-)	(-)	2.451 × 10^3^	4.149 × 10^3^	1.01 × 10^4^	(-)	1.239 × 10^4^	(-)	^††^ 1.903 × 10^3^
**8**	(-)	(-)	(-)	(-)	(-)	(-)	(-)	(-)	^††^ (-)
**9**	(-)	(-)	3.497 × 10^3^	6.703 × 10^3^	(-)	3.36 × 10^3^	(-)	(-)	^†^ (-)
**10**	(-)	(-)	(-)	(-)	(-)	(-)	(-)	(-)	3.051 × 10^3^
**DPI**	Sera (RNA copies/mL)								
**0**	(-)	(-)	(-)	(-)	(-)	(-)	(-)	(-)	(-)
**1**	(-)	(-)	(-)	(-)	1.455 × 10^3^	1.589 × 10^3^	(-)	2.253 × 10^3^	(-)
**3**	(-)	(-)	2.157 × 10^3^	(-)	9.549 × 10^2^	(-)	9.206 × 10^2^	(-)	(-)
**7**	(-)	(-)	(-)	(-)	(-)	(-)	(-)	(-)	(-)

DPI: Day(s) post-inoculation; (-): not detected; y: year; m: month; * Animal which presented episodes of diarrhoea. Diarrhoea severity: ^†^ mild; ^††^ moderate; ^†††^ severe. Limit of detection for RTqPCR assay is of 4.4 × 10^2^ RNA copies/mg or mL [44]. Animal weights at 3 dpi.
